# Safety and efficacy comparison between OACs plus single antiplatelet and dual antiplatelet therapy in patients with cerebral venous sinus stenosis poststenting

**DOI:** 10.1186/s12883-022-02731-0

**Published:** 2022-06-06

**Authors:** Chaobo Bai, Zhiying Chen, Xiaoqin Wu, Roxanne Ilagan, Yuchuan Ding, Xunming Ji, Ran Meng

**Affiliations:** 1 Department of Neurology, Xuanwu Hospital, Capital Medical University, Beijing, 100053 China; 2 Advanced Center of Stroke, Beijing Institute for Brain Disorders, Beijing, 100053 China; 3 Department of Neurology, Peking University Sixth Hospital, Peking University Institute of Mental Health, Beijing, China; 4 National Clinical Research Center for Mental Disorders, Peking University Sixth Hospital, Beijing, China; 5 Department of Neurosurgery, Wayne State University School of Medicine, Detroit, MI USA; 6 Department of Neurosurgery, Xuanwu Hospital, Capital Medical University, Beijing, 100053 China

**Keywords:** Cerebral venous sinus stenosis, Poststenting, Oral anticoagulants, Dual antiplatelet

## Abstract

**Background and purpose:**

The present strategies regarding poststent management for cerebral venous sinus stenosis (CVSS) are inconsistent. Herein, we compared the safety and efficacy of oral anticoagulants (OACs) plus single antiplatelet therapy and dual antiplatelet therapy for CVSS poststenting.

**Methods:**

A real-world observational study conducted from January 2009 through October 2019 enrolled patients who were diagnosed with CVSS and received stenting. Patients were divided into two groups according to the management they received poststenting. Group 1: OACs plus a single antiplatelet agent (clopidogrel 75 mg or aspirin 100 mg) and Group 2: dual antiplatelet therapy (clopidogrel 75 mg plus aspirin 100 mg). The safety (such as major or minor bleeding or venous thrombosis) and efficacy (the incidences of cerebral venous sinus restenosis, intrastent thrombosis, or stent displacement) of the two groups were compared.

**Results:**

There were a total of 110 eligible patients in the final analysis, including 79 females and 31 males with a mean age of 43.42 ± 13.23 years. No major bleeding or venous thrombosis occurred in either of the two groups. Two minor bleeding events occurred in group 2 (one with subcutaneous bleeding points in both lower limbs, another with submucosal bleeding in the mouth), whereas no bleeding events occurred in Group 1. In addition, at the 1-year follow-up, one case of intraluminal restenosis and two cases of in-stent thrombi occurred in Group 2, while none occurred in Group 1. Neither stenosis at stent-adjacent segments nor stent migration was detected in either group during the 1-year following stent placement.

**Conclusion:**

OACs plus single antiplatelet therapy and dual antiplatelet therapy alone are both safe and efficacious management strategies after CVSS stent placement. The former may have more advantages than the latter for inhibiting intrastent thrombosis. However, further research by larger, multicenter clinical trials is needed.

## Introduction

Studies on cerebral venous sinus stenosis (CVSS)-induced venous outflow obstruction have recently attracted attention [1–4]. Severe intracranial hypertension, in particular, has been discussed, as it is the vital culprit of CVSS and can cause a series of clinical symptoms and long-term irreversible neurological disabilities [1–3]. A number of treatment strategies have been explored, such as weight loss, cerebrospinal fluid secretion inhibitors, therapeutic lumbar puncture, and cerebrospinal fluid shunts; however, none of these strategies can obtain satisfactory clinical outcomes [5–7]. Despite this, there is now increasing evidence that venous stenting can significantly alleviate intracranial hypertension and eliminate clinical symptoms by rehabilitating venous outflow [8–10].

With the prevalence of stent placement for CVSS, treatment poststenting has become an emerging clinical topic, although no criteria about this issue have been established at present. Few studies have reported that the complication rate of dual antiplatelet therapy is lower than that of single antiplatelet therapy for CVSS poststenting [9, 11]. Other studies used warfarin combined with single antiplatelet therapy as the management strategy for CVSS poststenting [10, 12, 13]. Herein, we aim to compare the safety and efficacy between oral anticoagulant (OAC) plus single antiplatelet therapy and dual antiplatelet therapy on CVSS poststenting.

## Methods

### Study design and participants

The Ethics Committee and Institutional Review Board approved this single-center real-world observational study. Patients with CVSS who received stenting were enrolled from January 2009 through October 2019. After signing the informed consent forms, patients were divided into two groups according to their poststenting management. Group 1 received either OACs (dose-adjusted warfarin to maintain an international normalized ratio [INR] between 2.0 and 3.0) or novel oral anticoagulants (NOACs), which included dabigatran 110 mg/bid or rivaroxaban 15 mg/qd. In addition, Group 1 received a single antiplatelet therapy consisting of either clopidogrel 75 mg/qd. or aspirin 100 mg/qd. Group 2 received dual antiplatelet therapy consisting of 75 mg clopidogrel plus 100 mg aspirin.

Patients with cerebral venous thrombosis (CVT) underwent anticoagulation prior to stenting and were included in Group 1 (anticoagulant plus antiplatelet). The choice of poststenting medication for other patients depended on their background, specifically if they were or were not hypercoagulable. Patients with hypercoagulable backgrounds entered the anticoagulant plus antiplatelet group (Group 1), while patients without hypercoagulable backgrounds were included in the dual antiplatelet group (Group 2). There were no medication restrictions for patients requiring certain drugs for their chronic health conditions. The safety (including complications of major or minor bleeding or venous thrombosis) and efficacy (the rates of CVSS restenosis, intrastent thrombosis, and stent displacement) of the two groups were compared.

Inclusion criteria: 1) Age 18–80 years; 2) CVSS confirmed by contrast-enhanced magnetic resonance venography (CE-MRV)/computed tomography venous imaging (CTV)/digital subtraction angiography (DSA); 3) Intraoperative measured mean pressure gradient (MPG) across the stenosis segment ≥8 mmHg; 4) Poor response to routine medication control; 6) Signed the informed consent form.

Exclusion criteria: 1) Intracranial mass occupation; 2) Allergy to contrast agent or inability to finish CVS venography and stenting; 3) Patients with life-threatening diseases, such as severe cardiovascular or respiratory disorders or malignancy; 4) Those with severe bleeding events; 5) Patients unable to take oral medications or allergies to direct oral anticoagulants or antiplatelet agents; and 6) Incomplete clinical data.

### Treatment

Patients with CVSS who matched the inclusion criteria were enrolled. Individuals received low-molecular-weight heparin subcutaneous injections as well as an intravenous infusion of mannitol prior to stent placement.

#### Manometry and stent placement

Patients were placed in the supine position and received routine skin disinfection in the bilateral groin and perineal area. Sterile towel sheets were placed, and local infiltration anesthesia was administered. After successful puncture of the femoral vein, 5F arterial sheaths and 8F venous sheaths were placed, and systemic heparinization was performed to finish diagnostic venography to locate the segment of stenosis. Manometry was then performed at both sites (distal and proximal) of the stenosed segment, and the pressure gap between both sites was obtained. Self-expanding Acculink stents were placed when the mean pressure gradient (MPG) was ≥8 mmHg. Angiogram was then performed to reconfirm that the stenotic segment had been successfully corrected by the stent. MPG was remeasured to compare with its baseline to evaluate the effect poststenting.

#### Poststenting management

After excluding stent-related intracranial hemorrhage by immediate dual-energy computed tomography poststenting, all patients received a low molecular weight heparin (0.6 ml, q12 h) subcutaneous injection and mannitol (125 ml/q6 h, intravenous infusion). Blood pressure, heart rate, and oxygen saturation were monitored for 3 days. Lumbar puncture opening pressure follow-up was performed on day 3 of poststenting. Then, patients underwent OAC plus single antiplatelet (clopidogrel 75 mg or aspirin 100 mg) or dual antiplatelet therapy (clopidogrel 75 mg plus aspirin 100 mg), which was continued for 12 months.

#### Follow-up

Outpatient follow-up included evaluation for clinical symptoms, specific neuroimaging features, and stent-related complications, such as bleeding and CVT events during the 1-year poststenting.

#### Clinical assessment

##### 1. Bleeding events

Major bleeding: 1) life-threatening bleeding in critical organs such as the brain, spinal cord, ocular region, retroperitoneum, or pericardial sites; 2) bleeding-related hemoglobin reduction was more than 20 g per liter or needed to be immediately transfused by two or more units of whole blood or red blood cells; and 3) bleeding in situ in surgical sites, such as endovascular therapy, which required further management and resulted in prolonged hospitalization or delayed recovery. Bleeding data were recorded from the initiation of the stent operation until the fifth half-life after the last dose of medication used. All patients received at least one dose of the medication, and all bleeding events from the intake of the first dose until 1 year poststenting were counted [14].

Minor bleeding: 1) did not affect vital signs and daily life, 2) did not induce severe physical damage, 3) needed medical care and decreased the dosage of anticoagulant and/or antiplatelet agents, and 4) only transiently impacted the patients, such as small-scale skin bleeding, bulbar conjunctival bleeding, or urethral bleeding [15].

##### 2. Venous thrombosis

1) CVT occurrence or recurrence, 2) deep venous thrombosis (DVT), 3) pulmonary artery embolism, 4) visceral venous thrombosis, and 5) endo-stent thrombosis [16].

##### 3. Intracranial pressure assessment

The severity of optic papillary edema was assessed according to the Frisen optic papillary edema grade criteria [17]. Intracranial pressure was defined as the lumbar puncture opening pressure. The pressure difference gradient across the stenosis segment was expressed by the MPG.

##### 4. Neuroimaging evaluation

**Fig. 1:**
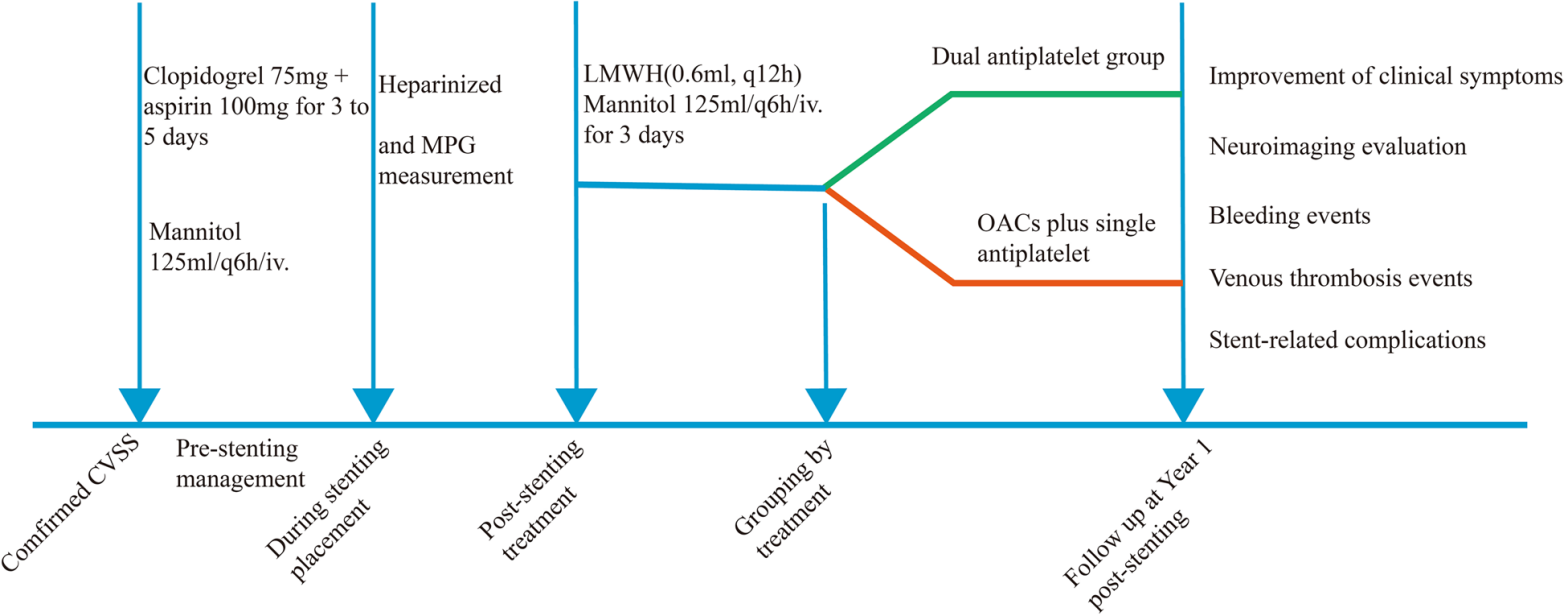
Design of drug flow chart of CVSS before and after treatment: LMWH indicates low-molecular-weight heparin. Dual antiplatelet group: clopidogrel 75 mg and aspirin 100 mg for 12 months. OACs group: aspirin 100 mg or clopidogrel 75 mg plus warfarin or NOACs for 12 months

3.0 T MRI maps were analyzed, including the sequences of axial T1WI, T2WI, DWI, FLAIR, ADC and CE-MRV. DSA was used to confirm CVSS and to obtain MPG. Two experienced radiologists analyzed the above imaging data. Details are displayed in Fig. 1**.**

### Statistical methodology

The Statistical Product and Service Solutions (SPSS) version 21.0 program (IBM, Armonk, NY: IBM Corp) was used for data analysis. The results were expressed as counts and percentages for categorical variables, as the mean ± standard deviation for continuous variables, and as the median (interquartile range) for discrete data. Fisher’s exact test was applied to evaluate differences in categorical variables, and Student’s *t* test was used to evaluate the differences in continuous variables. For data that did not meet the normal distribution, the Mann–Whitney U test was applied. Two-sided *p value*s <  0.05 were defined as statistically significant for all tests.

## Results

### Baseline data

Demographic data are shown in Table 1. A total of 110 eligible patients finished the 1-year follow-up poststenting: 52 patients were included in group 1, the oral anticoagulants plus antiplatelet group (female/male = 39/13), and 58 patients were included in group 2, the dual antiplatelet group (female/male = 40/18). Clinical manifestations between the two groups showed no significant differences regarding age, sex, BMI, tinnitus, visual decline, or papilledema; however, the ratio of headache showed a significant difference (65.4% vs. 31%), *p* <  0.001. Additionally, no statistical significance in comorbidities such as cerebral venous sinus thrombosis (CVST), type-2 diabetes mellitus, hypertension, coronary heart disease, and venous sinus stenosis were found (all *p* > 0.05). Details are displayed in Table 1.

**Table 1:** Baseline data in patients with CVSS post-stenting

Items	OACs plus antiplatelet (*n* = 52)	Dual antiplatelet (*n* = 58)	*p*-value
Demographic data			
Age	44.69 ± 12.89	41.38 ± 13.69	0.74
Gender (female/male)	39/13	40/18	0.48
Mean BMI (kg/m^2^)	26.20 ± 4.18	26.61 ± 3.98	0.79
Clinical manifestations			
Headache	34 (65.4%)	18 (31%)	< 0.001
Tinnitus	18 (34.6%)	11 (19%)	0.06
Visual decline	31 (59.6%)	36 (62.1%)	0.79
Papilledema	34 (65.4%)	46 (79.3%)	0.12
Comorbidities			
CVST	6 (11.5%)	3 (5.2%)	0.22
Type 2 diabetes mellitus	3 (5.8%)	2 (3.4%)	0.56
Hypertension	19 (36.5%)	13 (22.4%)	0.10
Hyperlipemia	6 (11.5%)	4 (6.9%)	0.40
Coronary heart disease	2 (3.8%)	3 (5.2%)	0.74
Side of CVSS			
TS	23 (44.2%)	29 (50%)	0.55
Superior sagittal sinus	2 (3.8%)	1 (1.7%)	0.46
Straight sinus	1 (1.9%)	1 (1.7%)	0.94
TS-SS boundary stenosis	32 (61.5%)	35 (60.3%)	0.90

Abbreviations: *CVSS* Cerebral venous-sinus stenosis, *CVST* Cerebral venous sinus thrombosis, *TS* Transverse sinus, *SS* sigmoid sinus

### Comparison of CVSS prior to and poststenting

All symptoms mentioned above were significantly diminished or even resolved poststenting, with statistical significance when compared to their baseline (*p* <  0.001). When comparing pre- vs. poststenting, Frisen scores were 3 (3–4): 2 (1–2), *p* <  0.001; lumbar puncture opening pressures were 293.44 ± 63.19 mmH_2_O vs. 183.89 ± 26.78 mmH_2_O, *p* <  0.001; and MPG was 8 (8.0–13.0) mmHg vs. 0.0 (0.0–0.0) mmHg, *p* <  0.001 (Table 2).

**Table 2:** Characteristics of patients with CVSS prior to and post-stenting

Items	Pre-stenting	Post-stenting	*p-value*
Symptoms			
Headache, n (%)	58 (47.5)	4 (3.3)	< 0.001
Tinnitus, n (%)	31 (25.4)	2 (1.6)	< 0.001
Visual decline	72 (59.0)	10 (8.2)	< 0.001
Papilledema, n (%)	85 (69.7)	49 (40.2)	< 0.001
FPG	3 (3–4)	2 (1–2)	< 0.001
ICP (mm water column)	293.44 ± 63.19	183.89 ± 26.78	< 0.001
MPG (mmHg)	8 (8.0–13.0)	0.0 (0.0–0.0)	< 0.001

Abbreviations: *CVSS* Cerebral venous-sinus stenosis, *FPG* Frisén papilledema grade, *ICP* Intracranial pressure, *MPG* Mean pressure gradient

### OACs plus single antiplatelet therapy vs. dual antiplatelet therapy poststenting

The outcomes of all 110 patients were compared, including 52 patients in the OAC plus antiplatelet group and 58 patients in the dual antiplatelet group (Table 3**).** Eight patients in the dual antiplatelet group were rejected (6 patients withdrew midway, and 2 patients had incomplete follow-up data), and 4 patients in the OAC group were rejected (the INR in 2 patients who underwent warfarin could not be maintained between 2 and 3, and 2 patients withdrew midway).

**Table 3:** One-year outcomes of the patients with CVSS post-stenting after underwent OACs plus antiplatelet or dual antiplatelet

Items	OACs plus antiplatelet (*n* = 52)	Dual antiplatelet (*n* = 58)	*p-value*
Major bleeding events, n (%)	0 (0.0)	0 (0.0)	NA
Minor bleeding events, n (%)	0 (0.0)	2 (3.4)	0.497
VTEs, n (%)	0 (0.0)	0 (0.0)	NA
Intraluminal restenosis	0 (0.0)	1(1.7)	1.000
In-stent thrombosis	0 (0.0)	2 (3.4)	0.497
Stent-adjacent stenosis	0 (0.0)	0 (0.0)	NA
Stent displacement	0 (0.0)	0 (0.0)	NA

Abbreviations: *OACs* direct oral anticoagulants, *VTEs* venous thrombotic events; Data were presented as n (%), mean ± SD or median (interquartile range). *NA* Not applicable

Two minor bleeding events occurred in the dual antiplatelet group, one case with gum bleeding and another with submucosal bleeding, while none were found in the OAC plus antiplatelet group. Neither major bleeding nor venous thrombotic events occurred in the two groups during the one-year follow-up poststenting. One case of intraluminal restenosis and 2 cases of in-stent thrombosis were present in the dual antiplatelet group; however, neither of them occurred in the OAC plus antiplatelet group. In addition, no stent-adjacent segment stenosis or stent migration was detected in the two groups (*p* > 0.05).

Subgroup analysis in the OAC plus antiplatelet group: The patients who received novel oral anticoagulants (19 cases of dabigatran and 11 cases of rivaroxaban) compared to warfarin (Table 4) had no major bleeding, minor bleeding, venous thrombosis, intrastent thrombosis, stent-adjacent stenosis, or stent-displacement (all *p* > 0.05).

**Table 4:** One-year outcomes of the patients with CVSS post-stenting after underwent single antiplatelet plus NOACs or warfarin

Items	NOACs (*n* = 30)	Warfarin (*n* = 22)	*p-value*
Major bleeding events, n (%)	0 (0.0)	0 (0.0)	NA
Non-major bleeding events, n (%)	0 (0.0)	0 (0.0)	NA
VTEs, n (%)	0 (0.0)	0 (0.0)	NA
Intraluminal restenosis	0 (0.0)	0 (0.0)	NA
In-stent thrombosis	0 (0.0)	0 (0.0)	NA
Stent-adjacent stenosis	0 (0.0)	0 (0.0)	NA
Stent displacement	0 (0.0)	0 (0.0)	NA

Abbreviations: *NOACs* Novel Oral Anticoagulants, *VTEs* venous thrombotic events; Data are presented as n (%), mean ± SD or median (interquartile range). *NA* Not applicable

## Discussion

### Treatment after stent placement to correct CVSS-induced severe intracranial hypertension is an important step

CVSS mainly involves the transverse sinus and the junction of the transverse sinus and sigmoid sinus, resulting in severe intracranial hypertension (ICP). Our results are consistent with other studies that demonstrated that stenting is an effective method for CVSS-induced severe intracranial hypertension correction [9, 18]. Although the MPG decreased poststenting, cerebral venous blood flow was restored, and the ICP was corrected immediately poststent management, which is still important for long-term favorable outcomes, and adequate treatment strategies are urgently needed [11, 19, 20].

### OACs plus antiplatelet therapy may be better than dual antiplatelet therapy for poststenting

There are no current guidelines about CVSS poststenting management. Some studies opted for aspirin plus clopidogrel 3–5 days prior to and 3–6 months poststenting, referring to the management in arterial disease [21–23]. One study used only clopidogrel as a poststenting treatment agent, and another three studies used oral warfarin for 8 weeks followed by a substitution to aspirin for 6 months or longer [10, 12, 13, 24]. There were several adverse events, such as in-stent thrombosis, in the studies using dual antiplatelet therapy for 3–6 months poststenting [21–23]. However, emulating the same treatment of arterial diseases may not be suitable because the venous internal environment differs when comparing atherosclerotic plaques in the vessel wall, blood components, and fit of the stent to the vessel wall [25]. Teleb et al. summarized 19 studies and noted that 2 out of 207 patients developed intrastent thrombosis. Fortunately, these patients achieved complete revascularization with adequate anticoagulation [26]. Our previous study revealed that the majority of patients with CVSS had a history of hypercoagulability, immune inflammation, thrombophilia, or thrombosis [27]. Although stenting could correct the local stenosis immediately, it could not correct their systemic thrombophilia. Moreover, based on follow-up using magnetic resonance venous thrombosis black blood imaging studies, long-term anticoagulation might be needed in CVSS poststenting [1–3]. Therefore, in this study, the duration of poststenting medical treatment was 1 year.

Previous studies have suggested that BMI is closely associated with idiopathic intracranial hypertension and may be a predictor of CVSS-related intracranial hypertension [4, 8, 28]. This conclusion was also found in our study, with a high proportion of overweight women in both groups. Cerebral venous outflow retardation induced by increased BMI is also a factor for consideration, as it also differs from arterial poststenting.

On the other hand, as a foreign body, the stent could provoke platelet overactivation, making antiplatelet agents necessary for prevention. Furthermore, it is well known that venous thrombosis differs from arterial thrombosis: arterial thrombi are platelet-rich and gather around ruptured atherosclerotic plaques and endothelial damage, while venous thrombi are mainly comprised of abundant fibrin, red blood cells, and a limited number of activated platelets [29]. Knowing this, antiplatelet therapy alone, as a strategy for venous thrombosis prevention poststenting, may not be enough. This information regarding the different pathological mechanisms of venous versus arterial thrombosis suggests that management for CVSS poststenting might differ from that for cerebral arterial poststenting management. Notably, this study suggests a lower rate of complications, especially restenosis, in both groups, which we speculate may be related to good patient compliance and an adequate course of treatment. However, more evidence is still needed for a more appropriate duration of treatment.

In this study, we compared the safety and efficacy of OACs plus single antiplatelet therapy with dual antiplatelet therapy and found that although there was no statistical significance between the two groups regarding bleeding events, venous thrombosis, intrastent thrombosis and other complications poststenting, there were still 2 cases of mild bleeding events, 1 case of intraluminal restenosis, and 2 cases of intrastent thrombosis in the dual antiplatelet group compared to none in the OAC plus antiplatelet group. Since there were no adverse events in the OAC plus single antiplatelet therapy group, OACs plus single antiplatelet therapy may have more promising effects; however, more evidence from a larger sample size is warranted to support this.

### Safety and efficacy of NOACs versus warfarin on CVSS poststenting

Warfarin is a commonly used anticoagulant agent. However, the genetic heterogeneity of its individual pharmacokinetic response, interaction with numerous foods and drugs, and requirement of regularly monitoring the international normalized ratio (INR) limit its use in the clinical setting. NOACs could specifically block certain coagulation factors (such as dabigatran for thrombin or rivaroxaban for factor Xa), thereby inhibiting the conversion of fibrinogen to fibrin, and have been proven to be as efficacious as warfarin for anticoagulation in some cardiac diseases, such as atrial fibrillation [30, 31]. A meta-analysis revealed that dabigatran was as efficacious as warfarin for preventing ischemic strokes in patients with nonvalvular atrial fibrillation and was associated with a lower risk of intracranial hemorrhage; however, it might promote gastrointestinal bleeding, especially in elderly individuals [32]. However, in this study, the majority of patients were young and middle-aged (mean age 43.42 ± 13.23 years), and no gastrointestinal bleeding events were observed.

A multicenter randomized controlled study compared the safety and efficacy of dabigatran and warfarin in preventing venous thrombotic events in patients with CVT and found that both dabigatran and warfarin were associated with lower risks of CVT recurrence and bleeding [16]. Consistent with previous studies, no bleeding events, venous thrombotic events, or stent-related complications were found in either the NOACs or warfarin groups, which suggested that NOACs may be as efficacious as warfarin for CVT control. The benefits of NOACs and the development of effective antagonists in recent years have led clinicians to favor NOACs [33, 34]. Given that NOACs do not require frequent monitoring of coagulation markers, this also improves patient compliance. Moreover, antagonists for adverse bleeding events are already available. The new oral anticoagulants may be more favored by clinicians and patients in clinical settings. However, multicenter, randomized clinical trials are still needed to provide more robust evidence.

### Limitations

First, this was only a single-center study. Multicenter studies with a large number of cases are still needed to further validate the conclusions. Second, the incidences of complications in CVSS poststenting were low in both groups, which might affect the assessment comparing OACs plus antiplatelet therapy and dual antiplatelet therapy. Another limitation in our study involved the choice to place patients with a higher risk of hypercoagulability in Group 1, thus making our two groups nonhomogeneous. In addition, we did not use genetic analysis for platelet drug resistance and could not assess whether it influenced the results. Our small sample size also failed to compare the differences among various NOACs. Another limitation was that the number of adverse events of stenting did not reach statistical significance. Finally, although the findings in this study provide a new reference for CVSS poststenting, long-term follow-up is still needed.

## Conclusion

Both OACs plus single antiplatelet therapy and dual antiplatelet therapy may be safe and efficacious for CVSS poststenting management. The former may have advantages compared to the latter for inhibiting intrastent thrombosis. However, further larger-scale studies are required to support these results.

## Data Availability

The raw data supporting the conclusions of this article will be made available by the corresponding author professor Ran Meng, without undue reservation.

## Abbreviations

CVSS: Cerebral venous sinus stenosis
OACs: Oral anticoagulants
CVT: Cerebral venous thrombosis

## References

[1] Li K, Ren M, Meng R, Ding Y, Rajah GB, Wang F, Ji X (2019). Efficacy of stenting in patients with cerebral venous sinus thrombosis-related cerebral venous sinus stenosis. J Neurointerv Surg.

[2] Xu Y, Meng R, Rajah GB, Ding Y, Wu Y, Wu Y, Ji K, Wu C, Zhao W, Ji X (2019). Long-term outcomes of cerebral venous sinus stenosis corrected by stenting. Curr Neurovasc Res.

[3] Bai C, Xu Y, Zhou D, Ding J, Yang Q, Ding Y, Ji X, Meng R (2019). The comparative analysis of non-thrombotic internal jugular vein stenosis and cerebral venous sinus stenosis. J Thromb Thrombolys.

[4] Peng K, Fuh J, Wang S (2012). High-pressure headaches: idiopathic intracranial hypertension and its mimics. Nat Rev Neurol.

[5] Mollan SP, Hoffmann J, Sinclair AJ (2019). Advances in the understanding of headache in idiopathic intracranial hypertension. Curr Opin Neurol.

[6] Mollan SP, Ali F, Hassan-Smith G, Botfield H, Friedman DI, Sinclair AJ (2016). Evolving evidence in adult idiopathic intracranial hypertension: pathophysiology and management. J Neurol Neurosurg Psychiatry.

[7] Skau M, Brennum J, Gjerris F, Jensen R (2006). What is new about idiopathic intracranial hypertension? An updated review of mechanism and treatment. Cephalalgia.

[8] Koovor JM, Lopez GV, Riley K, Tejada J (2018). Transverse venous sinus stenting for idiopathic intracranial hypertension: safety and feasibility. Neuroradiol J.

[9] Radvany MG, Solomon D, Nijjar S, Subramanian PS, Miller NR, Rigamonti D, Blitz A, Gailloud P, Moghekar A (2013). Visual and neurological outcomes following endovascular stenting for Pseudotumor Cerebri associated with transverse sinus stenosis. J Neuroophthalmol.

[10] Higgins JNP (2003). Idiopathic intracranial hypertension: 12 cases treated by venous sinus stenting. J Neurol Neurosurg Psychiatry.

[11] Ahmed RM, Wilkinson M, Parker GD, Thurtell MJ, Macdonald J, McCluskey PJ, Allan R, Dunne V, Hanlon M, Owler BK (2011). Transverse sinus stenting for idiopathic intracranial hypertension: a review of 52 patients and of model predictions. Am J Neuroradiol.

[12] Rajpal S, Niemann DB, Turk AS (2005). Transverse venous sinus stent placement as treatment for benign intracranial hypertension in a young male. J Neurosurg Pediatr.

[13] Higgins JNP, Owler BK, Cousins C, Pickard JD (2002). Venous sinus stenting for refractory benign intracranial hypertension. Lancet.

[14] Schulman S, Angerås U, Bergqvist D, Eriksson B, Lassen MR, Fisher W (2010). definition of major bleeding in clinical investigations of antihemostatic medicinal products in surgical patients. J Thromb Haemost.

[15] Ezekowitz MD, Connolly S, Parekh A, Reilly PA, Varrone J, Wang S, Oldgren J, Themeles E, Wallentin L, Yusuf S (2009). Rationale and design of RE-LY: randomized evaluation of long-term anticoagulant therapy, warfarin, compared with dabigatran. Am Heart J.

[16] Ferro JM, Coutinho JM, Dentali F, Kobayashi A, Alasheev A, Canhão P, Karpov D, Nagel S, Posthuma L, Roriz JM (2019). Safety and efficacy of dabigatran Etexilate vs dose-adjusted warfarin in patients with cerebral venous thrombosis. Jama Neurol.

[17] Frisen L (1982). Swelling of the optic nerve head: a staging scheme. J Neurol Neurosurg Psychiatry.

[18] Takkar A, Goyal MK, Bansal R, Lal V (2018). Clinical and neuro-ophthalmologic predictors of visual outcome in idiopathic intracranial hypertension. Neuro-Ophthalmology.

[19] Bai C, Chen J, Wu X, Ding Y, Ji X, Meng R (2020). Perioperative mannitol intensive use may avoid the early complication of cerebral venous sinus stenting. Ann Translat Med.

[20] Fields JD, Javedani PP, Falardeau J, Nesbit GM, Dogan A, Helseth EK, Liu KC, Barnwell SL, Petersen BD (2013). Dural venous sinus angioplasty and stenting for the treatment of idiopathic intracranial hypertension. J Neurointerv Surg.

[21] Liu KC, Starke RM, Durst CR, Wang TR, Ding D, Crowley RW, Newman SA (2017). Venous sinus stenting for reduction of intracranial pressure in IIH: a prospective pilot study. J Neurosurg.

[22] Levitt MR, Albuquerque FC, Ducruet AF, Kalani MYS, Mulholland CB, McDougall CG (2016). Venous sinus stenting for idiopathic intracranial hypertension is not associated with cortical venous occlusion. J Neurointerv Surg.

[23] Ducruet AF, Crowley RW, McDougall CG, Albuquerque FC (2014). Long-term patency of venous sinus stents for idiopathic intracranial hypertension. J Neurointerv Surg.

[24] Donnet A, Metellus P, Levrier O, Mekkaoui C, Fuentes S, Dufour H, Conrath J, Grisoli F (2008). Endovascular treatment of idiopathic intracranial hypertension: clinical and radiologic outcome of 10 consecutive patients. Neurology.

[25] Jakimovski D, Zivadinov R (2020). Use of patient-reported data in determining factors contributing to internal jugular vein stenosis outcomes. Ann Transl Med.

[26] Teleb MS, Cziep ME, Lazzaro MA, Gheith A, Asif K, Remler B, Zaidat OO (2013). Idiopathic intracranial hypertension: a systematic analysis of transverse sinus stenting. Intervent Neurol.

[27] Song S, Lan D, Wu X, Ding Y, Ji X, Meng R (2021). Clinical characteristics, inflammation and coagulation status in patients with immunological disease-related chronic cerebrospinal venous insufficiency. Ann Translat Med.

[28] Fargen KM, Liu K, Garner RM, Greeneway GP, Wolfe SQ, Crowley RW (2018). Recommendations for the selection and treatment of patients with idiopathic intracranial hypertension for venous sinus stenting. J Neurointerv Surg.

[29] Koupenova M, Kehrel BE, Corkrey HA, Freedman JE (2017). Thrombosis and platelets: an update. Eur Heart J.

[30] Bai Y, Deng H, Shantsila A, Lip GYH (2017). Rivaroxaban versus dabigatran or warfarin in real-world studies of Stroke prevention in atrial fibrillation. Stroke.

[31] Nagarakanti R, Ellis CR (2012). Dabigatran in clinical practice. Clin Ther.

[32] Romanelli RJ, Nolting L, Dolginsky M, Kym E, Orrico KB (2016). Dabigatran versus warfarin for atrial fibrillation in real-world clinical practice. Circulation.

[33] Aguiar De Sousa D, Lucas Neto L, Canhão P, Ferro JM (2018). Recanalization in cerebral venous thrombosis. Stroke.

[34] Ferro JM, Canhao P, Stam J, Bousser MG, Barinagarrementeria F (2004). Prognosis of cerebral vein and dural sinus thrombosis: results of the international study on cerebral vein and Dural sinus thrombosis (ISCVT). Stroke.

